# Feeding and Husbandry Practices in Pet Ferrets (*Mustela putorius furo*): A Cross‐Sectional International Study

**DOI:** 10.1002/vms3.71170

**Published:** 2026-08-13

**Authors:** Erdem Danyer, Giada Morelli, Lorenzo Serva, Elena Dal Corso, Diego Cattarossi, Marianne Diez, Rebecca Ricci

**Affiliations:** ^1^ Department of Animal Medicine Production and Health University of Padua, Padua Legnaro Italy; ^2^ Department of Biostatistics Faculty of Veterinary Medicine Dokuz Eylül University İzmir Türkiye; ^3^ Clinica Veterinaria Casale sul Sile Casale sul Sile Treviso Italy; ^4^ Department of Veterinary Management of Animal Resources University of Liège Liège Belgium

**Keywords:** body condition, exotic pets, ferret feeding, husbandry, survey, welfare

## Abstract

**Background:**

The domestic ferret (*Mustela putorius furo*) is an increasingly popular non‐traditional companion animal; however, limited information is available on how caregivers manage feeding and husbandry practices.

**Objectives:**

This study aimed to investigate feeding and management practices in pet ferrets and to evaluate differences among countries.

**Methods:**

A cross‐sectional, multilingual (Italian, English, French) online survey data collected from 495 ferret caregivers, mainly from France, Italy, the United States, the United Kingdom and Belgium.

**Results and Conclusions:**

Most ferrets were young adults, neutered (70.7%) and housed indoors (87.3%). Neutering practices differed significantly among countries, with surgical sterilisation more common in the United States and the United Kingdom (*p* < 0.001). Husbandry practices generally included daily free‐roaming and environmental enrichment; however, undesirable behaviours were frequently reported, particularly inappropriate elimination (79.0%) and food hiding (75.4%). Preventive veterinary care was common, although vaccination and parasite prevention rates were lower in the United Kingdom and the United States compared to continental Europe (*p* < 0.001). Feeding practices were highly variable. Dry commercial diets were the most commonly used (72.1%) and were typically offered ad libitum, while homemade diets were provided by 44.2% of caregivers and whole prey by 25.9%. Inconsistencies included use of products formulated for other species. Overall, husbandry and feeding practices varied considerably across countries. The wide variability in feeding approaches, inconsistent preventive care and lack of evidence‐based nutritional guidelines may increase the risk of nutritional imbalances and health issues, highlighting the need for clearer recommendations and improved caregiver education.

## Graphical abstract

Clearer recommendations and improved caregiver education are urgently needed to support optimal ferret welfare.

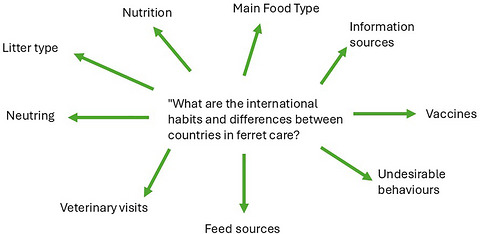

## 1 Introduction

The domestic ferret (*Mustela putorius furo*) is a non‐traditional companion animal, widely distributed across Europe and North America (Blanchard et al. 2018; Köbrunner et al. 2020; Powers and Perpiñán 2020; Stockman and Petritz 2024; Vinke and Schoemaker 2012).

Previous estimates suggest that the pet ferret population may reach up to ∼500,000 in the United States in 2018 (AVMA 2018), 300,000 in France (Vinke and Schoemaker 2012), 200,000 in the United Kingdom in 2022 (UK Pet Food 2026) and 105,000 in Italy (Vinke and Schoemaker 2012). However, reliable global estimates are lacking, as regulations vary widely between countries and registration of pet ferrets is often voluntary (Powers and Perpiñán 2020); therefore, available data are likely to underestimate the true population size. Although the ferret population has fluctuated over time, feeding practices, husbandry and welfare remain critical concerns, irrespective of population size.

The welfare of pet ferrets may be compromised when socialisation and husbandry practices, including housing, environmental enrichment, healthcare and feeding, are inadequate (Vinke and Schoemaker 2012; Harris 2015; Reijgwart et al. 2016). Environmental enrichment plays a key role in preventing behavioural problems. Previous studies have demonstrated that higher levels of enrichment, combined with reduced confinement time, are associated with fewer undesirable behaviours, such as bite injuries (Talbot et al. 2014). Common enrichment strategies for ferrets include the provision of hammocks, conspecific companionship, foraging opportunities and large water bowls (Reijgwart et al. 2016). In addition, appropriate feeding is essential, as it directly influences multiple aspects of ferret health and welfare.

Exotic animal specialists and veterinary nutritionists are frequently consulted by caregivers regarding optimal housing conditions and dietary management for pet ferrets. However, limited data are available on the extent to which the behavioural and nutritional needs of these animals are actually met (Bell 1999; Campbell‐Ward 2012; Church 2007b; Fox et al. 2014; Johnson‐Delaney 2014; Sá et al. 2014; Stockman and Petritz 2024).

More recent surveys have investigated husbandry practices adopted by caregivers, primarily focusing on health status and the occurrence of behaviours indicative of adequate or impaired welfare (Blanchard et al. 2018; Dancer et al. 2022a; Köbrunner et al. 2020; Talbot et al. 2014). For example, a study conducted among German‐speaking ferret owners identified several potential stressors and welfare concerns, including single housing (4%), bite injuries from conspecifics (4.6%), ferret‐specific diseases such as cardiac disease and adrenal tumours (6% each), and recent signs of illness such as diarrhoea (7.1%) or parasitic infections (6.2%). In addition, behaviours potentially affecting the human–animal relationship, such as defecation outside the litter area (reported daily in 27% of cases), were commonly observed (Köbrunner et al. 2020). These findings highlight the need for a more comprehensive understanding of housing conditions and nutritional practices in pet ferrets, as well as their potential impact on animal welfare.

Ferrets are obligate carnivores, similar to domestic cats (Stockman and Petritz 2024). Accordingly, diets high in protein and fat and low in carbohydrates, primarily derived from animal sources, are recommended, in line with feline nutritional requirements (Ball 2006; Bell 1999; Carpenter et al. 2010; Johnson‐Delaney 2014; Stockman and Petritz 2024).

A variety of commercial diets formulated for ferrets are currently available (Johnson‐Delaney 2014); however, homemade diets (HMDs), including raw meat‐based diets (RMBDs), have also gained popularity among caregivers (Morelli et al. 2021). Despite this, comprehensive and evidence‐based nutritional guidelines for ferrets remain limited, and the optimal diet for this species is still not well defined (Carpenter et al. 2010). Current knowledge on ferret nutrition is largely extrapolated from their obligate carnivorous physiology and supplemented by clinical experience and anecdotal evidence from caregivers and veterinarians (Torkelson et al. 2022).

Despite recognition of the ferret's carnivorous nature, a gap persists between nutritional requirements, diet‐related diseases and actual feeding practices. Previous surveys have only partially addressed nutritional trends and associated health outcomes, although the combination of commercial diets and fresh ingredients appears to be common among caregivers (Blanchard et al. 2018; Dancer et al. 2022b, Dancer et al. 2022a; Köbrunner et al. 2020; Talbot et al. 2014; Torkelson et al. 2022). Ferrets fed exclusively commercial diets or mixed diets (commercial and fresh food) have been reported to show a higher occurrence of lethargy, insulinoma, dental disorders, diarrhoea and ‘bird‐seed’ stools in contrast to those fed fresh food alone (Blanchard et al. 2018).

In addition, a wide range of dental pathologies has been described in ferrets, including tooth loss, bone erosion at the gingival margin, dental crowding, congenital absence of teeth, dental necrosis, abrasion associated with cage biting or object chewing, dental abscesses, calculus accumulation, fractures, palatine bone erosion and reactive bone formation (Church 2007b).

Although prevalence studies are lacking, overweight and obesity may represent emerging concerns in ferrets, based on clinical observations (Stockman and Petritz 2024). Seasonal increases in feed intake (up to 30% during winter) have been described and should not be misinterpreted as obesity (Bell 1999; Stockman and Petritz 2024). Hepatic lipidosis represents another serious metabolic disorder, particularly in ferrets affected by gastrointestinal disease, and may lead to severe outcomes, including sudden death in cases of hepatic rupture (Chitty 2023).

Considering the variability in feeding and husbandry practices reported in previous studies, there is a clear need for comprehensive, internationally comparative research. Therefore, the aim of this study was to characterise feeding and management practices of pet ferrets across different countries.

## 2 Materials and Methods

### 2.1 Participant Recruitment and Survey Design

A cross‐sectional study design was used. Between December 2020 and March 2021, ferret caregivers were voluntarily recruited through dedicated social media platforms to complete an online questionnaire. The questionnaire was initially developed in Italian using Google Forms and subsequently translated into English and French. A back‐translation method was applied to ensure accuracy and consistency across language versions (Sperber 2004). Prior to distribution, the questionnaire was pilot tested by the authors to assess clarity, consistency and comprehensibility. Data obtained during the pilot phase were excluded from the final analysis. To ensure internal validity, each participant was allowed to submit the questionnaire only once, corresponding to a single ferret.

The questionnaire consisted of 85 multiple‐choice questions and three open‐ended questions (Supporting Information File 1, English version). It was structured into four main sections:

1. *Caregiver demographics*: Age, gender, country of residence, previous experience with ferrets, household composition and employment in the animal care field.
2. *Ferret demographics*: Number of ferrets in the household, acquisition method, presence of other animals, age, coat characteristics, sex, neutering status and age at neutering, neutering method, body weight and length, body condition score (BCS; as assessed by the caregiver), sleep duration, activity level, faecal characteristics, temperament and general health status.
3. *Husbandry and management*: Housing conditions, time spent outside the cage, cage characteristics, litter type and environmental enrichment (e.g., toys, bowls and water dispensers). Additional questions addressed husbandry routines, including grooming practices (coat and ear cleaning, nail trimming), human–animal interactions, training history, frequency of veterinary visits, use of preventive treatments for parasitic and viral diseases, diagnosed conditions, dental care (professional or at‐home), sources of information on ferret care and the occurrence of specific behaviours.
4. *Nutrition*: Type of diet provided (e.g., commercial dry and wet food, HMDs and RMBDs), as well as the use of snacks and supplements, occurrence of adverse food reactions and water management practices. Participants were also asked to provide detailed information on feeding practices according to the type of diet(s) offered.

### 2.2 Used Likert Scales and Derived Variables

Participants were asked to score their ferrets’ coat characteristics (shininess, density, alopecia, dandruff and greasiness) using a five‐point Likert scale. Shine and density were scored from 1 to 5, whereas the absence of alopecia, dandruff and greasiness could be indicated with a score of 0.

Caregivers were also asked to report their perception of their ferret's BCS. This information was subsequently compared with reference body weight ranges reported by Powers and Perpiñán (2020), according to sex and neutering status (intact males: 1.0–2.0 kg; intact females: 0.6–0.95 kg; neutered males and females: 0.8–1.2 kg).

Based on caregiver‐reported sex, neutering status and body weight, a categorical variable was generated to classify ferrets as underweight, ideal weight or overweight.

### 2.3 Sample Size Calculation

The study was initially designed to investigate the pet ferret population in Italy and France; therefore, the sample size was calculated based on these two countries.

The ferret population in Italy and France was estimated at 405,000 animals (Vinke and Schoemaker 2012). Assuming an expected frequency of 0.5, a confidence limit of 0.06, a confidence level of 95% and a design effect of 1, the required sample size was calculated to be 267 using the below equation (Dean et al. 2024).

$$n=\mathrm{deff}\times \frac{N\hat{p}\hat{q}}{\frac{d^{2}}{1.96^{2}}\left(N-1\right)+\hat{p}\hat{q}}$$

$$\begin{array}{c} \mathrm{where}\;n=\;\mathrm{sample}\;\mathrm{size},\;\mathrm{deff}=\;\mathrm{design}\;\mathrm{effect},\;N=\\ \;\mathrm{Total}\;\mathrm{Italian}\;\mathrm{and}\;\mathrm{French}\;\mathrm{estimated}\;\mathrm{ferret}\;\mathrm{population}, \end{array}$$

$$\begin{array}{ccc} & & \;\hat{p}=\\ & & \;\mathrm{expected}\;\%\;\mathrm{frequency}\;\mathrm{of}\;\mathrm{outcome}\;\mathrm{factor}\;\mathrm{in}\;\mathrm{the}\;\mathrm{population},\\ & & \;\;\hat{q}=1-\hat{p},\;d=\;\mathrm{confidence}\;\mathrm{limits}\;\mathrm{as}\;\%\;\mathrm{of}\;100\;\left(\mathrm{absolute}\;\pm \%\right) \end{array}$$

### 2.4 Statistical Analysis

Data collected from the questionnaire were transferred to a spreadsheet (Microsoft Excel). No imputation was performed for missing values, and analyses were conducted using the original responses. Quantitative and qualitative variables were summarised as frequencies and percentages (*n*/*N*; %). Countries contributing less than 5% of total responses were grouped under the category ‘other countries’. The distribution of continuous variables was assessed for normality. Parametric data were analysed using Student's *t*‐test, whereas non‐parametric data were analysed using the Mann–Whitney *U* test. For each variable, observed frequencies were compared among countries (France, Italy, the United Kingdom, the United States and Belgium) using the chi‐squared test, implemented in R (version 4.0.5). Responses from ‘other countries’ were included in overall analyses but excluded from between‐country comparisons. When no significant differences were detected among the main countries, data were pooled for subsequent analyses. Statistical significance was set at *p* < 0.05.

## 3 Results

### 3.1 Survey Distribution and Validity

A total of 495 ferret caregivers voluntarily completed the questionnaire across multiple countries (Table 1). The category ‘other countries’ included responses from Australia (1/495, 0.2%), Canada (2/495, 0.4%), Croatia (1/495, 0.2%), Denmark (3/495, 0.6%), Germany (1/495, 0.2%), Ireland (4/495, 0.8%), Malaysia (1/495, 0.2%), Slovenia (10/495, 2.0%), Spain (1/495, 0.2%), Sweden (1/495, 0.2%) and Switzerland (2/495, 0.4%). The target sample size for Italy and France (*n* = 267) was achieved, with these countries accounting for 70.3% of responses (348/495).

**TABLE 1:** Demographic characteristics of survey participants (*n* = 495).

Parameter	Variable	Ferret caregivers, *n* (%)
Country	France	186 (37.6)
	Italy	162 (32.7)
	US	68 (13.7)
	UK	39 (7.9)
	Belgium	13 (5.5)
	Other countries	27 (8.1)
Gender	Male	50 (10.1)
	Female	444 (89.7)
	Other	1 (0.2)
Age (years)	< 18	11 (2.2)
	18–25	134 (27.1)
	26–35	165 (33.3)
	36–45	108 (21.8)
	46–55	48 (9.7)
	56–65	22 (4.4)
	> 65	7 (1.4)
Household composition	Single	55 (11.1)
	Couple	193 (39.0)
	Family	214 (43.2)
	Other	33 (6.6)
Household members at risk of zoonotic exposure	Children < 4 years old	29 (6.5)
	Children 4–14 years old	94 (21.0)
	Elderly > 65 years old	33 (7.4)
	Pregnant women	11 (2.5)
	Immunocompromised person	42 (9.4)
	None	268 (59.8)

### 3.2 Caregiver Demographics

Participant characteristics are summarised in Table 1. Most respondents were women (444/495, 89.7%), aged 18–35 years (270/495, 54.5%) and living with a partner or within a family setting (407/495, 82.2%). Nearly half of households (217/495, 43.8%) included at least one individual at increased risk of zoonotic infections, such as children, elderly individuals, pregnant women or people with chronic illness or immunocompromised conditions. No significant differences were observed among countries in the proportion of households with at‐risk individuals (*p* = 0.17). At the time of data collection, 48.5% of participants (240/495) reported previous experience as ferret caregivers. A small proportion of respondents were ferret breeders (14/495, 2.8%), veterinary medicine students (12/495, 2.4%) or veterinarians (6/495, 1.2%).

### 3.3 Ferret Demographics

Ferret demographics are reported in Tables 2 and 3. Most caregivers owned two or more ferrets (324/495, 65.5%) and other companion animals (322/495, 65.5%), including dogs (210/495, 42.4%), cats (205/495, 41.4%), small mammals (44/495, 8.8%), reptiles (35/495, 7.0%) and birds (25/495, 5.0%). Over the previous 2 months, 26.2% of ferrets had been housed with one or more conspecifics (85/324). Most ferrets were young adults (i.e., 2–5 years old; 274/495, 55.3%) and were perceived by caregivers to have an ideal body condition (463/495, 93.5%). Males were slightly overrepresented in contrast to females (296/495, 59.8% vs. 199/495, 40.2%) (Table 2).

**TABLE 2:** Demographic characteristics of ferrets included in the study (*n* = 495).

Parameters	Variable	Ferrets, *n* (%)
Number of ferrets per household	1	171 (34.5)
	2	164 (33.1)
	3	63 (12.7)
	> 3	97 (19.6)
Gender and neutering status		
Female	Intact	48 (9.7)
	Neutered	151 (30.5)
Male	Intact	97 (19.6)
	Neutered	199 (40.2)
Age	< 6 months	9 (1.8)
	6–12 months	111 (22.4)
	2–3 years	171 (34.5)
	4–5 years	103 (20.8)
	6–8 years	81 (16.4)
	> 8 years	8 (1.6)
	Missing values	12 (2.4)
Caregiver reported body condition	Underweight	11 (2.2)
	Normal weight	463 (93.5)
	Overweight	21 (4.2)
BCS according to Powers and Perpiñán (2020)	Underweight	72 (14.5)
	Normal weight	226 (45.7)
	Overweight	132 (26.7)
	Not available data	65 (13.1)

**TABLE 3:** Caregivers’ reported body weight (kg) and body length (cm) of ferrets (*n* = 495). Data are reported as mean ± standard deviation (minimum–maximum).

Parameters		*n* (%)	Body weight	Body length
Female Male	Intact	48 (9.7)	0.94 ± 0.39 (0.16–2.00)	40.22 ± 11.27 (17.00–60.00)
	Neutered	151(30.5)	0.87 ± 0.40 (0.25–3.50)	40.53 ± 11.24 (15.00–65.00)
	Intact	97 (19.6)	1.60 ± 0.53 (0.45–3.20)	48.33 ± 14.77 (15.00–80.00)
	Neutered	199 (40.2)	1.40 ± 0.46 (0.45–3.50)	44.79 ± 11.95 (16.00–70.00)

Most ferrets were neutered (350/495, 70.7%), with surgical and chemical sterilisation being similarly represented (195/350, 55.7% and 155/350, 44.3%, respectively). However, significant differences were observed among countries (*p* < 0.001), with surgical sterilisation being far more common in the United States (67/69, 98.5%) and the United Kingdom (36/39, 91.3%) than in France (101/186, 54.0%), Belgium (5/13, 37.5%) and Italy (46/162, 28.4%). The mean age at sterilisation varied by country: 18 ± 17 months (range: 6–48) in Belgium, 14 ± 13 months (range: 3–72) in France, 14 ± 14 months (range: 3–72) in Italy and 15 ± 13 months (range: 6–60) in the United Kingdom. In contrast, ferrets in the United States were neutered at a significantly younger age (2 ± 1 months; range: 1–6) compared with those in other countries (*p* < 0.001).

Mean body weight differed significantly between males (1.45 ± 0.48 kg) and females (0.88 ± 0.39 kg) (*p* < 0.001). A significant difference was also observed between intact and neutered males (*p* = 0.002), whereas no difference was detected between intact and neutered females (Table 3). Regarding coat characteristics, most ferrets had short hair (333/495, 67.3%), with sable (136/495, 27.5%) and black sable (70/495, 14.1%) being the most common coat colours, and a standard pattern reported in 46.9% of cases (232/495). When caregivers were asked to score coat characteristics using a 0–5 Likert scale (shininess, density, alopecia, dandruff and greasiness), most ferrets were reported to have moderate to high coat shininess (scores 3–5; 73.3%) and density (77.7%). Alopecia and dandruff were infrequently reported, with 86.4% and 90.9% of ferrets receiving a score of 0, respectively. Regarding greasiness, 63.2% of ferrets were reported to have no greasiness (score 0), while 21.4% received a score of 1.

### 3.4 Housing Conditions of Ferrets

The majority of ferrets (432/495, 87.3%) were reported to live indoors full‐time; 8.7% (43/495) were kept indoors only for part of the day, while 4.0% (20/495) lived exclusively outdoors. Most ferrets (314/495, 63.4%) were housed in cages with free access to exit. These animals were allowed to move either freely throughout the house (189/495, 38.2%) or within a designated area (125/495, 25.3%).

A proportion of ferrets were not housed in cages: 16.6% (82/495) were allowed to roam freely throughout the house at all times, while 14.3% (71/495) had a room entirely dedicated to them. Only a small proportion of ferrets (8/495, 1.6%) were permanently confined to a cage.

Excluding ferrets that were always free‐roaming or not housed in cages (121/495, 24.4%), those with limited access outside the cage were allowed out for more than 8 h/day (102/495, 20.6%), 4–8 h/day (146/495, 29.5%), 2–4 h/day (86/495, 17.4%), 1–2 h/day (31/495, 6.3%) or less than 1 h/day (9/495, 1.8%). Approximately, one in five caregivers did not provide a cage for their ferrets (98/495, 19.8%), whereas the majority selected multi‐level cages (369/495, 74.5%).

The most commonly reported environmental enrichment items included litter trays (461/495, 93.1%), food bowls (471/495, 95.2%), water bowls (407/495, 82.2%), drinking bottles (169/495, 34.1%), hammocks (422/495, 85.3%), hiding places (389/495, 78.6%), ladders or ramps (309/495, 62.4%), toys (334/495, 67.5%) and tunnels or mazes (250/495, 50.5%). Less frequently reported items included dig boxes (90/495, 18.2%), ropes (55/495, 11.1%), blankets (34/495, 6.9%) and beds (29/495, 5.9%).

Most ferrets appeared to prefer drinking from bowls (317/495, 64.0%) rather than from bottles (64/495, 12.9%). Most caregivers reported changing the water once daily (285/495, 57.6%) or more than once per day (133/495, 26.9%), while a smaller proportion changed it less than once per day (70/495, 14.1%).

The most commonly used litter types were clumping litter (118/495, 23.8%), plant‐based materials (e.g., corncob; 115/495, 23.2%), paper (92/495, 18.6%), pellets (85/495, 17.2%) and puppy training pads (61/495, 12.3%). Clumping litter was most frequently reported in Italy (45/162, 27.8%) and France (45/186, 24.2%), whereas plant‐based materials were more common in France (68/186, 36.6%) and Belgium (4/13, 30.8%). Paper litter was most frequently used in the United States (29/68, 44.1%) and the United Kingdom (10/39, 25.6%), while pellets were more common in Belgium (4/13, 30.8%) and the United States (18/68, 26.5%). Puppy pads were more frequently used in the United Kingdom (9/39, 23.1%) and Italy (37/162, 22.8%) (*χ* ^2^ = 249.3; *p* < 0.001).

Most caregivers reported changing the litter once daily (158/495, 31.9%) or more than once per day (118/495, 23.8%), while others reported changing it every other day (84/495, 17.0%), once weekly (65/495, 13.1%), twice weekly (62/495, 12.5%) or less than once per week (8/495, 1.6%).

Nail trimming was the most regularly performed grooming practice, with 49.1% of caregivers (243/495) reporting that they performed it twice per month. In contrast, coat cleaning was the least frequently performed routine, with over one‐third of caregivers (38.2%, 189/495) reporting that they never carried it out. Ear cleaning showed a more variable pattern, with most caregivers performing it once per month (25.1%, 124/495), followed by those doing it less than once per month (20.4%, 101/495) and those who never performed it (20.2%, 100/495).

When asked how much time the caregivers spent interacting with their ferrets in a day, they mostly stated 2–4 h/day (194/495, 39.2%), 1–2 h/day (133/495, 26.9%), more than 4 h/day (105/495, 21.2%); some stated 0.5–1 h/day (56/495, 11.3%) or less than 0.5 h/day (7/495, 1.4%).

Most ferrets were reported to sleep for more than half of the day, specifically 16–20 h/day (219/495, 44.2%) or 12–16 h/day (159/495, 32.1%). Only a small proportion were reported to sleep more than 20 h/day (29/495, 5.9%), while the remaining ferrets slept 8–12 h/day (78/495, 15.8%) or less than 8 h/day (10/495, 2.0%). When assessing the level of activity, the caregivers described their ferrets as very active (67/495, 13.5%), active (374/495, 75.6%), calm (51/495, 10.3%) or inactive (3/495, 0.6%).

The following undesirable behaviours were noticed by owners as having occurred either recently or in the past: defecating or urinating outside the litter tray (391/495, 79.0%), food hiding (373/495, 75.4%), compulsive scratching (186/495, 37.6%), biting or attacking other people (149/495, 30.1%) or the caregiver (122/495, 24.6%), pacing along the same path (118/495, 23.8%), biting or attacking objects or the cage (82/495, 16.6%), biting or attacking other ferrets (78/495, 15.8%) and self‐biting or self‐injury (4/495, 0.8%). Only 4.6% of the caregivers (23/495) reported never observing any of these behaviours.

When the occurrence of undesirable behaviours was analysed in relation to housing characteristics, food hiding was reported in 49.8% (241/484) of ferrets housed in enclosures with ladders or ramps (*p* = 0.006). The prevalence increased to 61.0% (295/484) in cages containing a hiding place (*p* = 0.02), and further to 71.1% (344/484) when a litter tray was present (*p* = 0.02).

The frequency of defecation and urination outside the litter tray was 66.9% (324/484) in cages with a water bowl (*p* = 0.002); the occurrence decreased to 54.3% (263/484) (*p* = 0.005) in the absence of a drip drinker. The frequency of inappropriate elimination decreased to 23.8% (115/484) when litter was changed daily (*p* = 0.02) and to 21.1% (102/484) in ferrets housed in multi‐level cages (*p* = 0.004).

The occurrence of biting or attacking the owner was 2.7% (13/484) in the presence of a dig box (*p* = 0.02) and, in contrast, the ratio of biting or attacking humans increased up to 24.2% (117/484) in ferrets housed in multi‐level cages (*p* = 0.02).

Some ferrets were trained by their caregivers to come when called (311/495, 62.8%), to use the litter (271/495, 54.7%) and to do exercises (104/495, 21.1%) whereas 17.2% of the caregivers (85/495) reported not training their ferrets.

### 3.5 Preventive Veterinary Care

Participants reported that 71.7% of ferrets (311/495) were regularly vaccinated, 16.2% (80/495) were vaccinated irregularly, and 12.1% (60/495) were not vaccinated. Regarding parasite prevention, 56.4% of caregivers (279/495) reported administering treatments regularly, while 22.6% (112/495) indicated irregular treatment and 21.0% (104/495) reported no parasite prevention. Specifically, 54.7% of ferrets (271/495) were regularly treated for heartworm prevention (caused by *Dirofilaria immitis*), 34.1% (169/495) were treated irregularly, and 11.1% (55/495) received no treatment.

The higher proportion of ferrets that were never vaccinated or treated for parasite and heartworm prevention lived in the United Kingdom and the United States. In particular, 53.8% and 23.5% of ferrets were never vaccinated in the United Kingdom and the United States, compared to the 0% in Belgium, 7.5% in France and 4.3% in Italy ($\chi^{2}$ = 133.6; *p* < 0.001); 38.5% and 63.2% of ferrets were never treated for parasite prevention in the United Kingdom and the United States, compared to the 7.7% in Belgium, 13.4% in France and 9.3% in Italy ($\chi^{2}$ = 171.9; *p* < 0.001); 84.6% and 75% of ferrets were never treated for heartworm disease prevention in the United Kingdom and the United States, compared to the 46.2% in Belgium, 60.8% in France and 32.7% in Italy ($\chi^{2}$ = 143.4; *p* < 0.001).

Most ferrets were taken to the veterinarian for routine check‐ups once (225/495, 45.5%) or twice (101/495, 20.4%) per year. Smaller proportions were taken more than twice per year (76/495, 15.4%) or less than once per year (6/495, 1.2%), while 17.6% (87/495) were presented only when health issues arose.

Ferrets living in Belgium and Italy were examined more frequently than those in other countries (*χ* ^2^ = 204.0; *p* < 0.001). In particular, more than 60% of ferrets in Belgium and over 50% in Italy were taken to the veterinarian at least twice per year, compared with less than 30% in France and approximately 20% in both the United Kingdom and the United States. This difference was statistically significant (*χ* ^2^ = 204.0; *p* < 0.001).

**FIGURE 1:**
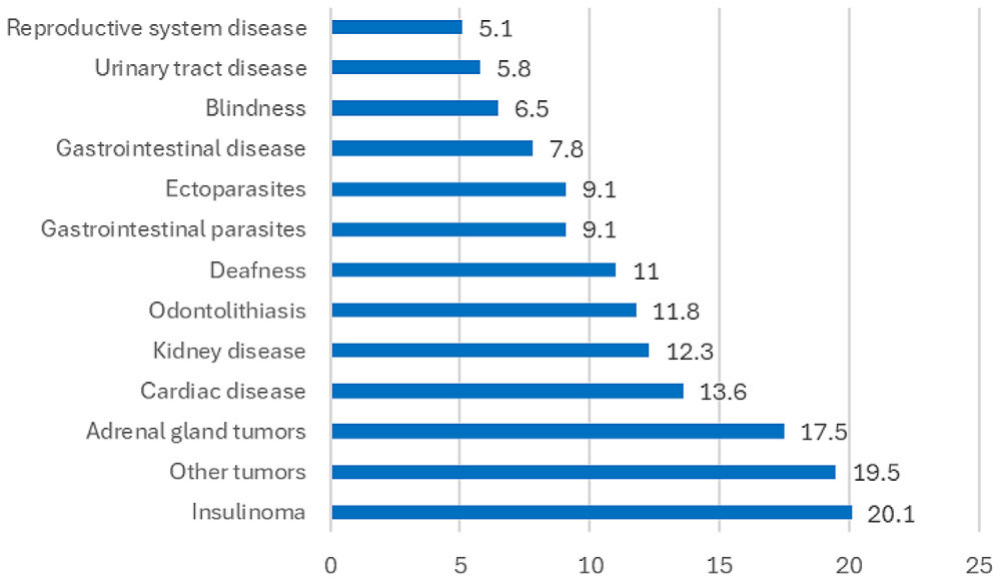
Distribution of diagnosed diseases in ferrets at the time of data collection (*n* = 154). Results are expressed as percentages.

Of the 495 ferrets included in the study, 86.3% (341/495) had no diseases reported by caregivers, whereas 13.7% (154/495) had at least one diagnosed condition at the time of data collection. The distribution of caregiver‐reported diagnoses is presented in Figure 1.

Adrenal gland tumour was mostly reported in ferrets from Belgium (2/13; 15.4%), the United States (8/68; 11.8%) and Italy (11/162; 6.8%) ($\chi^{2}$= 40.7; *p* = 0.03). Ferrets diagnosed with adrenal gland tumours (*n* = 21) were neutered at a mean age of 12.2 ± 16.2 months (*n* = 14), whereas those without adrenal gland tumours were neutered at a mean age of 10.8 ± 12.3 months (*n* = 293) (*p* = 0.71). Quarter of the participants discerned an unusual texture of their ferret's faeces (liquid: 1/495, 0.2%; soft: 128/495, 25.9%; hard: 1/495, 0.2%). Some caregivers noticed the presence of alopecia on their ferrets (67/495, 13.5%), mainly visible on the tail (58/495, 11.7%) and on the trunk (9/495, 1.8%).

Almost all ferrets included in this study had never undergone dental cleaning procedures (455/495, 91.9%), such as tartar removal or tooth brushing, although a minority of caregivers (68/495, 13.7%) acknowledged the need for dental care. Only a small proportion of ferrets had undergone professional tartar removal by a veterinarian, either once (24/495, 4.8%) or more than once (16/495, 3.2%) during their lifetime. No association was found between dry food and reported dental tartar cases (*n* = 18) in ferrets (*p* = 0.82). The majority of caregivers did not routinely brush their ferret's teeth (425/495, 85.9%). Among those who reported brushing, the frequency was generally low: less than once per week (37/495, 7.5%), once per week (23/495, 4.6%), every other day (7/495, 1.4%) or more than once per day (3/495, 0.6%).

Owners were asked to identify their most reliable source of information regarding ferret healthcare, general management and nutrition. Overall, 53.9% (266/495) considered veterinarians to be the most reliable source for healthcare information. Among these, the majority referred specifically to veterinarians specialised in exotic animal medicine (203/495, 41%). In contrast, only 16.0% (79/495) of caregivers considered veterinarians to be the most reliable source of information on housing management, a proportion lower than that reported for websites (121/495, 24.4%) and associations (86/495, 17.4%). For nutrition and feeding, the most frequently reported sources of information were veterinarians specialised in exotic animal medicine (107/495, 21.6%) and social media groups (97/495, 19.6%) (Table 4).

**TABLE 4:** Primary sources of information on ferret care among participants (*n* = 495). Results are expressed as number of respondents and percentage.

Source	Healthcare	Husbandry	Nutrition
Associations	42 (8.5)	86 (17.4)	68 (13.7)
Books	13 (2.6)	20 (4.0)	9 (1.8)
Exotic animal veterinarian	203 (41.0)	63 (12.7)	107 (21.6)
Ferret breeders	24 (4.8)	42 (8.5)	47 (9.5)
Groups on social media	70 (14.1)	67 (13.5)	97 (19.6)
Other ferret caregivers	30 (6.1)	57 (11.5)	36 (7.3)
Veterinarian	64 (12.9)	16 (3.2)	34 (6.9)
Online sources	38 (7.7)	121 (24.4)	70 (14.1)
Other	11 (2.2)	23 (4.6)	27 (5.5)

Most ferrets were acquired either through an association or from a previous caregiver (159/495, 32.1%), from a private individual (145/495, 29.3%) or from a breeding facility (116/495, 23.4%). In contrast, only a minority were purchased from pet shops (69/495, 13.9%).

### 3.6 Feeding Management of Ferrets

The majority of caregivers fed their ferrets dry food (357/495, 72.1%), either as the sole diet (181/357, 50.7%) or in combination with HMDs (133/357, 37.3%) or wet commercial food (76/357, 21.3%). Wet food was provided by 16.8% of caregivers (83/495), either as the sole diet (4/83, 4.8%) or in combination with HMDs (36/83, 43.3%). Overall, 44.2% of caregivers (219/495) reported feeding HMD, which was used as the sole diet in 37.9% of cases (83/219). A small proportion of respondents (33/495, 6.6%) reported providing all three types of food. Among caregivers providing dry food, 40.9% (146/357) fed dry food specifically formulated for ferrets, 40.3% (144/357) used cat food and 18.2% (65/357) reported using both ferret‐specific and other companion animal dry food (e.g., for dogs and cats). However, when participants were asked to report the brand and commercial name of the products used, 10.4% (37/357) indicated a product formulated for a different species than previously stated (e.g., several respondents who reported feeding ferret‐specific diets were in fact using cat food). Most caregivers reported never changing the brand or type of feed (275/357, 77.0%). A smaller proportion changed it once or twice per year (44/357, 12.3%), whereas 4.8% (17/357) reported changing it three to six times annually. More frequent changes were less common, including weekly (9/357, 2.5%), monthly (8/357, 2.2%) or biweekly (4/357, 1.1%) changes.

Almost all caregivers (336/357, 94.1%) provided feed ad libitum, while only a minority offered controlled (18/357, 5.0%) or variable (3/357, 0.8%) amounts. Among those providing controlled portions, feed quantities were most commonly estimated visually (11/18, 61.1%), followed by the use of a measuring cup (4/18, 22.2%) or a kitchen scale (2/18, 11.1%). Dry feed was continuously available for most ferrets (338/357, 94.7%), while a small proportion received meals at fixed (11/357, 3.1%) or variable times (8/357, 2.2%).

Caregivers feeding dry food were more likely to report compact faecal scores (*χ* ^2^ = 16.2; *p* = 0.001; 365/495). Ferrets fed dry food were also more frequently reported to bite conspecifics (*χ* ^2^ = 4.5; *p* = 0.04; 64/78) and to hide food (*χ* ^2^ = 9.2; *p* = 0.003; 256/373).

Among caregivers providing wet commercial food, 15.7% (13/83) used products specifically formulated for ferrets, 66.3% (55/83) used cat food and 15.7% (13/83) reported using both. However, when participants were asked to report the brand and commercial name of the products used, 20.5% (17/83) indicated a product intended for a different species than previously stated. Most caregivers feeding wet food reported never changing the brand or type (56/83, 67.5%), while others reported changing it weekly (9/83, 10.8%) or biweekly (3/83, 3.6%). Less frequent changes included monthly (4/83, 4.8%), once or twice per year (6/83, 7.2%) or three to six times annually (5/83, 6.0%). In contrast to dry feeding practices, most caregivers provided controlled (64/83, 77.1%) or variable (12/83, 14.5%) amounts of wet food, whereas only a minority offered it ad libitum (7/83, 8.4%). Among those providing controlled portions, quantities were primarily estimated visually (54/64, 84.4%), followed by the use of a measuring cup (5/64, 7.8%) or a kitchen scale (5/64, 7.8%). Wet food was continuously available for only a small proportion of ferrets (6/83, 7.2%), while most received meals at fixed (49/83, 59.0%) or variable times (28/83, 33.7%). Ferrets fed wet food were more frequently reported to defecate or urinate outside the litter tray (58/391, 14.8%; *χ* ^2^ = 5.4; *p* = 0.02) and to bite their caregivers (12/122, 9.8%; *χ* ^2^ = 4.9; *p* = 0.03).

**FIGURE 2:**
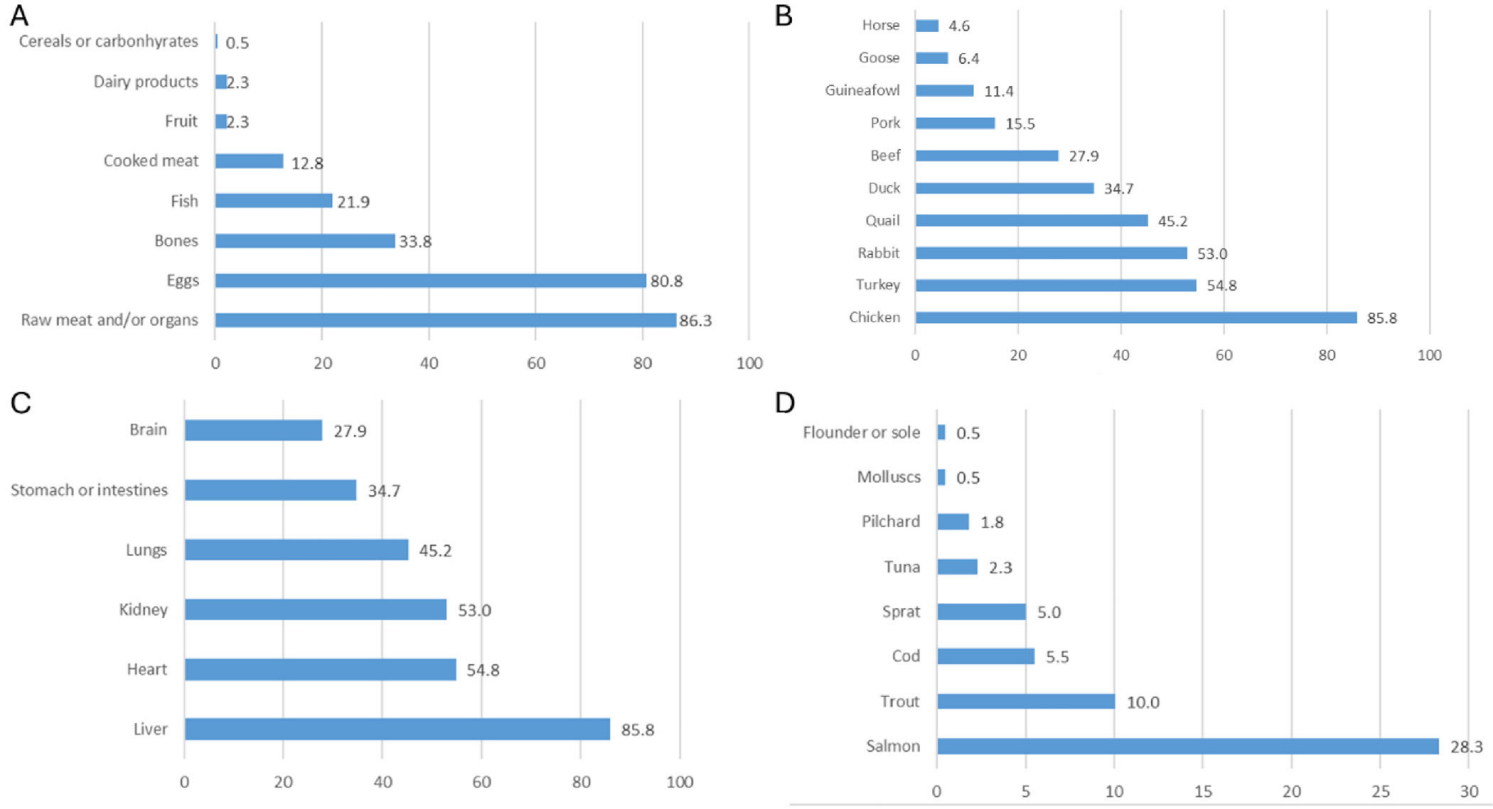
The most common (A) fresh foods, (B) meat types, (C) offal and (D) fish provided by 219 participants giving HMDs to their ferrets. Results are expressed as percentages.

Nearly half of the ferrets (219/495, 44.2%) were fed HMDs, either exclusively or in combination with commercial diets (wet and/or dry). Figure 2A–D illustrates the most commonly used fresh food items. Meat, organs and eggs were the most frequently included ingredients, with chicken, turkey and rabbit being the most commonly used meat sources. Among organ meats, liver and heart were the most widely used. In addition, 43.4% (95/219) of caregivers reported regularly including fish, predominantly salmon and trout. A total of 92 caregivers provided an estimate of the weekly distribution of fresh food components (Figure 2).

**FIGURE 3:**
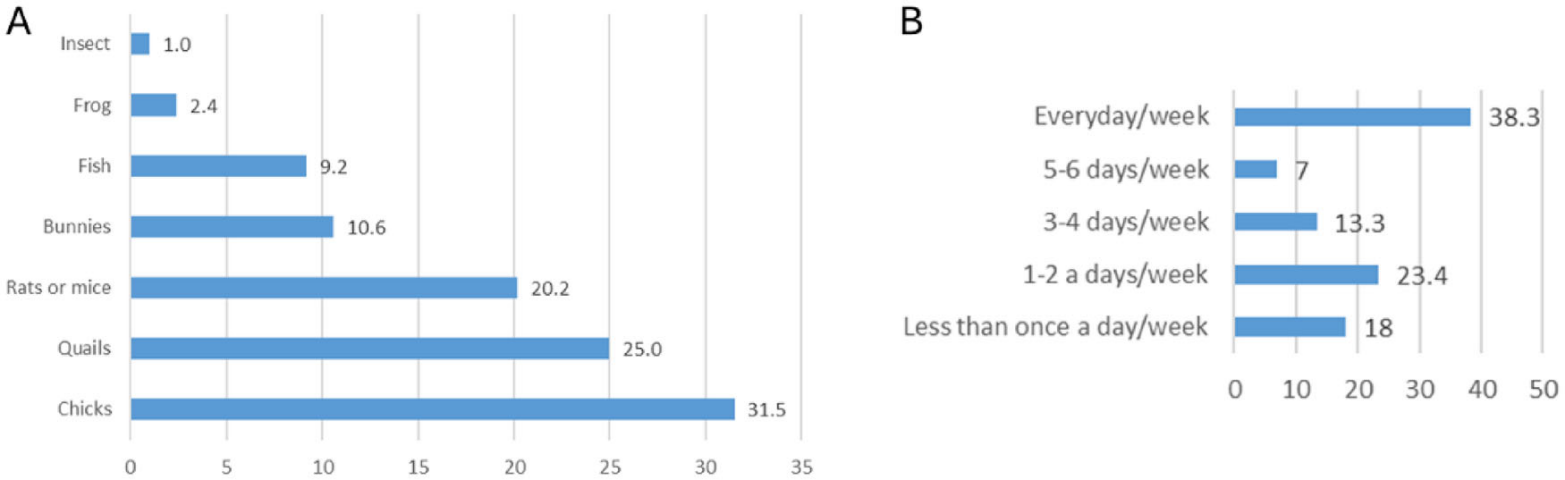
The most common (A) whole preys (*n* = 292) fed by the participants and (B) their providing frequency (*n* = 100). Results are expressed as percentages.

Most caregivers provided controlled (145/219, 69.4%) or variable (21/219, 10.0%) amounts of fresh HMD, while a smaller proportion offered it ad libitum (43/209, 20.6%). Among those providing controlled portions, quantities were most commonly estimated visually (80/145, 55.2%), followed by the use of a kitchen scale (51/145, 35.2%) or a measuring cup (14/145, 9.7%). Fresh food was continuously available for a minority of ferrets (31/209, 14.8%), whereas more than half received meals at fixed times (114/209, 54.5%) or at variable times throughout the day (64/209, 30.6%). A quarter of participants (128/495, 25.9%) reported feeding whole prey, most commonly chicks, quails and small rodents. Among these, 38.3% (49/128) provided whole prey daily (Figure 3). Ferrets fed HMD were more frequently reported to hide food (182/373, 48.8%; *χ* ^2^ = 12.7; *p* < 0.001), as were those fed whole prey (106/373, 28.4%; *χ* ^2^ = 5.2; *p* = 0.03).

Snacks were provided by 71.3% of participants (353/495), including commercially available treats (116/495, 23.4%), homemade treats (92/495, 18.6%) or a combination of both (141/495, 28.5%); only four participants (0.8%) reported offering complete feed as a snack. The most commonly used snack items included meat and organ products, eggs and treats intended for other companion animals. Among those providing snacks, 25.4% (90/353) offered them once daily, while 17.5% (61/353) did so more than once per day.

Half of the participants (248/495, 50.1%) reported including dietary supplements in their ferrets’ diets. The most commonly used products were those aimed at improving coat condition, followed by vitamin and mineral supplements. However, caregivers providing skin‐related supplements or fish oil were not more likely to report higher coat quality scores in their ferret (*p* > 0.05).

No adverse food reactions were reported by 79.4% of caregivers (393/495). Among the remaining respondents, the most commonly reported triggers were chicken (26.3%), commercial pet foods (25.3%), eggs (20.2%) and organ meats (16.2%).

Differences in fresh food provision by country (including only countries with more than 13 responses) are reported in Table 5. All data can be seen in Supporting Information File 2. Notably, caregivers in the United Kingdom reported lower use of RMBDs, as well as reduced inclusion of organs, bones and eggs, with no reported use of fish or dairy products. Chicken, turkey and rabbit were the most commonly used meat sources in Italy and the United States, whereas pork and beef were more frequently reported in the United Kingdom. In Belgium, duck, pork and rabbit were the most commonly used meats. Liver and heart were the most frequently included organ meats across all countries. Whole prey items (e.g., chicks, mice and rats) were also used, particularly in the United Kingdom. Commercial treats formulated for ferrets and malt paste were commonly used in Belgium and Italy, respectively. Dietary supplements, including those aimed at improving coat quality, supporting gastrointestinal health or providing vitamins and minerals, were also used, with varying prevalence across countries.

**TABLE 5:** Fresh foods, snacks and supplements provided to ferrets enrolled in the study (*n* = 495), stratified by country. Results are expressed as percentage.

	Belgium	France	Italy	UK	US
RMBDs and organs	30.8	35.5	44.4	23.1	30.9
Eggs	38.5	38.2	33.3	23.1	32.4
Bones	15.4	11.8	14.8	10.3	11.8
Dairy products	0.0	0.5	1.9	0.0	0.0
Meat types					
Chicken	38.5	35.5	42.6	25.6	30.9
Duck	30.8	12.4	11.7	17.9	19.1
Rabbit	30.8	17.7	30.9	15.4	17.6
Offal					
Liver	23.1	23.7	26.5	12.8	22.1
Heart	30.8	18.3	29.0	20.5	23.5
Fish					
Salmon	23.1	19.4	2.5	2.6	16.2
Preys					
Chicks	30.8	28.0	6.2	30.8	5.9
Mice or rats	15.4	13.4	3.7	33.3	5.9
Quails	0.0	12.4	19.1	12.8	7.4
Snacks					
Industrial snacks for ferrets	53.8	22.0	10.5	10.3	27.9
Malt paste	0.0	9.7	45.1	2.6	2.9
Supplements					
Coat	23.1	30.1	26.5	7.7	19.1
Gastrointestinal	15.4	8.1	22.8	5.1	2.9
General vitamin and mineral	23.1	13.4	35.8	10.3	11.8

## 4 Discussion

The accurate assessment of ferret population trends remains challenging, largely due to the limited implementation of mandatory microchipping regulations. In many countries, microchipping is required only for ferrets involved in cross border travel (European Union 2013; USDA 2025), which likely contributes to underestimation of the true population size. Data from the US Pet Ownership and Demographic Sourcebooks indicate a decline in the proportion of pet ferrets among companion animals in the United States, from 0.5% in 2001 to 0.1% in 2021 (AVMA 2022; Shepherd 2008).

During the early coronavirus disease 2019 (COVID‐19) pandemic, ferrets were identified as susceptible to severe acute respiratory syndrome coronavirus 2 (SARS‐CoV‐2) (Kim et al. 2020; Shi et al. 2020), leading to media reports describing the removal of ferrets from households (BBC 2020). Consequently, the Welsh Government introduced registration measures for mustelids (Wales 2021). While full evaluations are pending, media coverage and the use of ferrets in scientific research may have contributed to shifted public perception and ownership trends of ferrets globally.

Despite the apparent decline in the ferret population, there remains a clear need to improve the collection, understanding and dissemination of information on nutritional and husbandry practices to ensure the welfare of this relatively novel companion animal. Nevertheless, a substantial knowledge gap persists regarding domestic feeding and management practices, highlighting the need for further research.

Responses’ distribution enabled the assessment of husbandry practices across both European and non‐European contexts and facilitated comparison with previous surveys. However, the availability of the questionnaire in a limited number of languages may have reduced participation from caregivers in other countries.

### 4.1 Caregiver Demographics

Caregiver characteristics are crucial to understand husbandry, nutritional and healthcare practices of the pet ferrets. The predominantly female participant profile (89.7%) is consistent with previous reports indicating that online questionnaires tend to attract more women than men (Wu et al. 2022). Although this proportion is high, similar gender distributions have been reported in other ferret studies (Dancer et al. 2022b; Köbrunner et al. 2020).

In this study, most respondents were younger than 35 years (62.6%, 310/495), in line with findings from a German survey in which 74.3% of participants (416/573) were under 35 years of age (Köbrunner et al. 2020). The majority of respondents reported living as part of a couple or family (82.2%, 407/495), supporting the role of ferrets as companion animals within household settings.

Ferrets may act as reservoirs for several zoonotic pathogens (Pignon and Mayer 2011; Zaffarano 2010) and this survey explored the potential risk of zoonotic exposure within households. Vulnerable groups, including children, older adults (> 65 years), pregnant individuals and immunocompromised persons, are known to be at increased risk of zoonotic infections (Stull et al. 2012). Nearly half of respondents (40.2%, 227/495) reported the presence of at least one at‐risk individual in the household. Future studies should address this aspect to better evaluate risk perception and management.

### 4.2 Ferret Demographics

Approximately 85.5% of respondents reported owning more than one ferret, which is consistent with recommendations to house ferrets in groups, as they are highly social animals (Graham and Knafo 2021).

Several characteristics of the study population were expected, including sexual dimorphism (i.e., higher body weight and length in males) and the higher proportion of neutered compared with intact ferrets. These findings are in agreement with previous surveys conducted in France, Germany and Australia (Blanchard et al. 2018; Köbrunner et al. 2020; Talbot et al. 2014).

Most ferrets (approximately 80%) were between 1 and 5 years of age, corresponding to young adults. Given that life expectancy in ferrets ranges from 5 to 11 years (Powers and Perpiñán 2020), older individuals were underrepresented in the study population, with only 17% aged 6–8 years and fewer than 2% older than 8 years.

In contrast, the proportion of ferrets undergoing chemical sterilisation was higher than expected. In the present study, 44.3% of ferrets were chemically sterilised, compared with 20.6% reported in France (Blanchard et al. 2018) and 9.1% in German‐speaking countries (Köbrunner et al. 2020). This discrepancy may reflect regional differences in veterinary practices and the increasing interest in non‐surgical sterilisation methods.

Chemical sterilisation in companion animals has been extensively investigated, with dogs representing the most studied species and considerable variation in adoption rates across countries (Romagnoli et al. 2024). Notably, Italy has emerged as a particularly active research hub in this field, with several studies specifically focusing on canine chemical sterilisation (Cicirelli et al. 2022; Leoci et al. 2014), which may have influenced its application in other species, including ferrets. Although country‐specific data on neutering methods in ferrets remain limited, the higher prevalence of chemical sterilisation observed in this study likely reflects differences in regional veterinary trends.

A significant difference in the age of neutering was also identified. In the United States, ferrets are commonly surgically sterilised at a very young age, as commercially bred animals are often neutered before sale or distribution (Powers and Perpiñán 2020). This practice likely accounts for the younger age at neutering observed in this population compared with other countries.

### 4.3 Housing Conditions of Ferrets

In this survey, 87.3% of ferrets lived exclusively or predominantly indoors, in agreement with previous studies reporting proportions of 86.1% and 76.1% (Blanchard et al. 2018; Köbrunner et al. 2020). Regardless of housing type, most ferrets were allowed additional free‐roaming time for several hours per day. As ferrets benefit from regular time outside their primary enclosure to explore and play (Powers and Perpiñán 2020), this aspect appears to be widely recognised by caregivers, both in the present study and in previous reports (Dancer et al. 2022a; Köbrunner et al. 2020; Talbot et al. 2014).

The results also suggest that housing conditions may influence the occurrence of undesirable behaviours. Certain environmental enrichment items, such as ladders, ramps and hiding places, were associated with increased food‐hiding behaviour, whereas dig boxes were associated with reduced biting, indicating that enrichment may both stimulate natural behaviours and modulate behavioural outcomes. In contrast, the presence of multi‐level cages and hammocks was associated with increased biting and inappropriate elimination, indicating that not all forms of cage structure are equally beneficial. These findings emphasise the significance of carefully balancing cage design and enrichment provision in order to support rather than impair ferret welfare.

Environmental enrichment is widely considered beneficial for the welfare of captive ferrets, as in other species (Dancer et al. 2022a; Vinke and Schoemaker 2012). Previous studies have identified enrichment strategies that align with ferrets’ behavioural needs, including opportunities for burrowing, tunnelling, digging, climbing and hiding (Powers and Perpiñán 2020; Vinke and Schoemaker 2012). Providing appropriate resting areas, such as enclosed sleeping spaces or hammocks, is also essential (Vinke and Schoemaker 2012).

However, ferrets appear to require highly variable environments (Bays et al. 2006), and enrichment items should therefore be regularly modified or rearranged. In the present study, most caregivers reported providing hammocks, hiding places, ladders or ramps, toys and tunnels or mazes. In contrast, Köbrunner et al. (2020) reported a higher diversity of enrichment items among caregivers in German‐speaking countries.

Evidence regarding the relationship between enrichment and undesirable behaviours remains inconsistent. Talbot et al. (2014) suggested that providing a high number of enrichment items (≥ 6) may increase arousal, which can manifest as both play behaviour and undesirable behaviours. In addition, certain items, such as narrow tunnels or chewable rubber objects, may pose safety risks due to entrapment or ingestion (Dancer et al. 2022a).

Although most caregivers provided water in bowls, consistent with the demonstrated preference of ferrets for open water sources (Köbrunner et al. 2020; Reijgwart et al. 2016), providing water in bowls may increase the risk of environmental moisture due to spillage. Therefore, recommending wide and shallow bowls to caregivers may reduce this risk.

Clumping litter was the most commonly used type, although some authors recommend paper‐based substrates to reduce potential health risks, as ferrets may occasionally ingest litter (Powers and Perpiñán 2020). Questions on litter type were not included in previous surveys; however, participants in the present study reported renewing litter several times per day (23.8%) or daily (31.9%). These frequencies were higher than those reported by Köbrunner et al. (2020) (several times per day, 4.6%; daily, 11.3%). Litter type likely depends on product availability across countries. In addition to conventional litter, puppy pads were commonly used, particularly in the United Kingdom and Italy, likely for ease of cleaning.

When comparing grooming practices and their frequency, participants in the present study reported cleaning their ferrets’ coats more frequently than those in Köbrunner et al. (2020) (‘never’: 38.2% vs. 73.7%; ‘> 1/month’: 12.9% vs. 0.4%). However, frequent bathing is discouraged in some countries, as it may negatively affect welfare (Köbrunner et al. 2020). Similarly, nail trimming was performed more frequently in the present study, with 59.4% of caregivers reporting trimming more than once per month (vs. 40.5% in Köbrunner et al. 2020). In contrast, ear cleaning was less frequently performed in the present study (‘never’: 10.2% vs. 20.2%; ‘> 1/month’: 25.2% vs. 34.3%).

Sleeping durations exceeding the typical range (14–18 h/day) have been suggested as a potential indicator of boredom in ferrets (Burn 2017; Dancer et al. 2022b). In the present study, only a small proportion of ferrets were reported to sleep more than 20 h/day (5.9%), suggesting a low prevalence of potential boredom. This interpretation is further supported by nearly 90% of caregivers describing their ferrets as active.

Many ferrets in this study were reported to exhibit behaviours perceived as undesirable, particularly defecation or urination outside the litter tray (79.0%), food hiding (75.4%) and repetitive scratching (37.6%). These behaviours were also commonly reported by Köbrunner et al. (2020), albeit at higher frequencies (83.3%–74.5%, 92.7% and 61.2%, respectively). Notably, inappropriate elimination remained frequent despite 54.7% of participants reporting litter training, supporting previous observations that house‐training in ferrets is often only partially successful (Bulloch and Tynes 2010). In addition, 44.3% of caregivers reported cleaning litter trays less than once daily, which may contribute to this behaviour, as ferrets are known to mark their territory, particularly when litter tray is not sufficiently clean (Vinke and Schoemaker 2012).

Some behaviours, such as pacing, repetitive scratching or cage biting, may indicate unmet behavioural needs and reduced welfare. However, these were reported at relatively low frequencies, lower than those observed in previous surveys (Köbrunner et al. 2020), suggesting that the behavioural needs of most ferrets in this study were likely met. Repetitive locomotor behaviours, such as pacing, were less frequently reported than repetitive scratching, in line with previous findings (Normando and Gelli 2011; Talbot et al. 2014).

### 4.4 Preventive Veterinary Care

Alongside appropriate housing, preventive healthcare is fundamental to ferret welfare. Results suggest regional differences in preventive healthcare practices for ferrets. Compared with participants from other countries, caregivers in the United Kingdom reported lower uptake of routine veterinary visits and vaccinations, indicating a greater reliance on reactive rather than preventive veterinary care.

It should be noted that rabies has been eradicated from terrestrial animal populations in the United Kingdom (Public Health England 2019), which may contribute to a lower perceived need for vaccination among caregivers. Similarly, parasite prevention was less frequently reported in the United Kingdom compared with France, Italy and Belgium, and 63.2% of respondents from the United States reported not performing parasite prevention. A comparable pattern was also observed for heartworm prevention.

As an island nation, the United Kingdom benefits from distinct geographical advantages that facilitate stricter control over cross‐border animal movements, which may contribute to limiting the risk of certain infectious diseases. According to notifiable diseases occurrence list for Great Britain and Northern Ireland (APHA 2026) and guidance document for imported disease summaries for dogs and cats (DEFRA 2022), the climatic conditions in the United Kingdom are generally unsuitable for the completion of the life cycle of *Dirofilaria* spp. Therefore, endemic establishment of this parasite is unlikely under current environmental conditions. Reported sporadic cases are primarily associated with the importation of infected animals rather than infestation in United Kingdom. Compared with many other European and Mediterranean countries, the overall risk of parasitic diseases in the United Kingdom remains relatively low (DEFRA 2022). This may explain the lower level of preventive measures observed in this study. However, despite the limited environmental suitability for certain vector‐borne parasites, preventive healthcare remains crucial. As highlighted by Vinke and Schoemaker (2012), routine preventive care against ectoparasites, endoparasites and infectious diseases such as canine distemper is essential for maintaining optimal health and welfare in ferrets. Even in regions with low endemic risk, preventive strategies should not be neglected, as animal movement and climate variability may alter disease dynamics over time.

Apart from vaccination and parasite control, the prevalence of common ferret diseases provides valuable data for assessing the efficacy of preventive healthcare in pet ferrets. Adrenal tumours and insulinomas are the most common endocrine diseases in ferrets, and a correlation has been found between neutering and the occurrence of adrenocortical tumours (Shoemaker 2009; Shoemaker et al. 2000). Contrary to expectations, the lack of a detectable correlation for adrenal diseases and young neutering may be attributable to the limited number of reported adrenal disease cases in the subsample. The French study carried out by Blanchard et al. (2018) reported a much higher prevalence of tartar (11%) and adrenal tumours (4.2%) than the current study (1.1% and 1.6%, respectively). The effect of dry food on ferret oral health is yet to be determined. Blanchard et al. (2018) found univariate correlations between dry diets, tartar and fractures, but these associations were not supported in multivariate models, possibly due to limited sample sizes or diagnostic and reporting limitations. This study also supports these findings by giving no definitive evidence of a dry food and reported dental tartar cases. Overall, the prevalence of pathologies identified in the current survey is comparable to that reported by Köbrunner et al. (2020) in Germany/Austria/Switzerland.

### 4.5 Feeding Management of Ferrets

Nutritional management, like preventive care, showed substantial variation among respondents and countries. Feeding a diet based on whole prey animals is supposed to be the most natural diet for ferrets (Carpenter et al. 2010; Church 2007a; Harris 2015; Powers and Perpiñán 2020). Three quarters of the participants did not offer whole prey animals to their ferrets, contrary to previous surveys in which such percentage was limited to 46% in France and 29% in Germany/Austria/Switzerland (Blanchard et al. 2018; Köbrunner et al. 2020). The present survey revealed that whole prey feeding, despite being considered the most biologically appropriate diet for ferrets, remains largely unpopular among ferret owners. As a result, HMDs and kibbles were more commonly reported as the most common nutritional approach among responders.

A combination of commercial and HMD was given by three participants out of four, and Blanchard et al. (2018) as well as Köbrunner et al. (2020) reported a similar finding in their surveys. Some authors encourage the feeding of mixed diets as an environmental enrichment for pet ferrets (Powers and Perpiñán 2020). However, Blanchard et al. (2018) reported that ferrets fed with commercial food only or with a mixed diet were more likely to suffer from certain diseases (e.g., insulinoma and diarrhoea), compared with ferrets fed with fresh food only in their study. Given the strict carnivore nature of the ferret, some authors recommend to lower the carbohydrate intake, either through the use of a balanced HMD or balanced commercial wet foods for kittens or ferrets (Blanchard et al. 2018).

HMD was provided in different combinations, and ingredients selection varied widely among countries. Plant‐based ingredients (e.g., cereals, vegetables and fruit) were almost never fed by the participants to this survey, but they were much more common in previous studies (Blanchard et al. 2018; Köbrunner et al. 2020). Generally, deficiencies of specific vitamins and minerals are more likely to occur in ferrets fed with poorly formulated HMD rather than commercial foods intended for cats (Carpenter et al. 2010). It seemed unlikely that ferret caregivers participating in this study relied on nutrition specialists to obtain complete and balanced recipes; in fact, it emerged that only 22% considered the exotic animal veterinarian the most reliable source on ferret nutrition. Since only 142/495 caregivers purchased supplements for balancing HMD or vitamin and mineral supplements, common micronutrient deficiencies in the recruited ferrets can be suspected.

When the use of treats was investigated, many caregivers considered meat and eggs as snacks, although commercially available treats were commonly used too; treats and malt paste were likewise very common in the survey by Köbrunner et al. (2020). Ferrets are reported to like sweets, therefore it is not a surprise that such products are particularly appreciated as snacks (Powers and Perpiñán 2020). However, some authors discourage the feeding of treats not to predispose ferrets to insulinomas (Powers and Perpiñán 2020). Since the exact nutritional requirements of pet ferrets remain unknown, recommendations for the most appropriate diet in this species have still not been adequately determined (Carpenter et al. 2010; Powers and Perpiñán 2020). Further studies are warranted to define precise nutritional requirements for pet ferret.

Obesity is a common health problem in pet animals (German 2010). Pet owners frequently underestimate the condition of overweight and obesity in their animals (Corbee 2014; Muñoz‐Prieto et al. 2018). In this study, the body condition of the ferrets was assessed based on the caregivers' perceptions. Subsequently, owner‐reported body weights were used, together with gender and sterilisation condition to estimate the BCS of the ferrets according to previous recommendations (Powers and Perpiñán 2020). While 93.5% of ferrets were reported to be in normal body condition according to their owners, only 52.6% were estimated to be in normal body condition based on the ranges reported by Powers and Perpiñán (2020). This discrepancy highlights the low reliability of owners in evaluating their pet's BCS, as it was previously demonstrated also with dogs and cats (Kienzle et al. 1998).

Previous studies carried out on non‐traditional small mammal companion animals highlighted that obese rabbits and rodents are at high risk for multiple metabolic diseases, including urinary tract disease, gastrointestinal dysbiosis and orthopaedic disease, similar to dogs and cats (Stockman and Petritz 2024). Monthly body condition and weight checks have been recommended to prevent obesity in ferrets (Vinke et al. 2018).

Generally, caregivers and practitioners are unfamiliar with ferret ideal body conditions. According to the weight classification by Powers and Perpiñán 2020, 30.7% of the ferret pets in this study were classified as overweight. While weight classification alone is not a comprehensive method for determining an animal's BCS, it can provide preliminary information when more detailed data are unavailable.

The results of the present study indicate a potential obesity risk presumably due to inappropriate nutrition and inactivity and high probability of metabolic and nutritionally caused diseases in ferrets. Given the wide range of dietary habits reported by ferret owners, healthcare professionals should keep a high level of suspicion for nutrition‐related diseases during routine health checks, as poor nutritional management may predispose ferrets to a variety of metabolic conditions.

The development of specialised feed formulations tailored to the specific energy requirements of ferrets emerges as a critical research priority in ferret nutrition. Such studies could significantly contribute to optimising nutritional management and overall health in this species.

Generally, the study indicates that ferret welfare is often maintained by dedicated caregivers, but nutritional management and inadequate preventive care are common vulnerabilities across countries. Owner‐reported BCS underestimate, limited use of health professionals and regional differences in preventive medicine all point to the need for standardised, species‐specific husbandry protocols.

Certain limitations in this study should be considered, in order to better interpret its results. First, the survey was shared only on one social media, and the participants were self‐selected, so there may have been a sampling bias (i.e., age and gender). All husbandry and health information were based on participants reports, which may be subject to recall bias. When defining the BCS, visual examples were not provided, and responses were collected based on categories such as underweight, normal weight and overweight. Although the responses exceeded the target sample size, the findings remain limited by the nature of the survey data and the timing of data collection. Given the length of the survey, it is possible that only the most motivated caregivers took the time to fill it out, and these people may be more concerned with their ferrets’ health and wellness than average. The peculiarity of every single diet (i.e., commercial, HMD and RMBD) was not investigated in detail, as this was not the aim of the study; however, it could be useful to examine them using ad hoc future questionnaires. Nonresponse bias was mitigated with the implementation of obligatory question controls in Google Forms.

## 5 Conclusions

In conclusion, the data from this survey suggest that most ferrets’ welfare and living conditions are conscientiously taken care of by their caregivers. Many differences may be found depending on the country, especially regarding healthcare and nutritional management. Various foods in different combinations are offered to ferrets, and the lack of precise guidelines along with the lower veterinary consultations rates for nutritional advice may pose risks for nutritional imbalances. Healthcare professionals can recommend trusted resources on good husbandry practices to owners in order to improve their knowledge. It is suggested that more research be undertaken on the ferret feeding, the energy and nutrient content of diets, as well as welfare needs.

## Author Contributions

G.M., L.S., E.D.C., D.C., M.D. and R.R. were responsible for the creation of the questionnaire. G.M., E.D.C., D.C. and M.D. shared the questionnaire and collected the responses. G.M., E.D. and L.S. performed the statistical analysis and interpreted the data. G.M. and E.D. drafted the manuscript. R.R., L.S., E.D.C., D.C. and M.D. revised the manuscript. All authors have read and agreed to the published version of the manuscript.

## Funding

Erdem Danyer was supported by the 2219 Fellowship Programme of the Scientific and Technological Research Council of Türkiye, Project No: 1059B192300618.

## Ethics Statement

The authors confirm that the ethical policies of the journal, as noted on the journal's author guidelines page, have been adhered to. No ethics approval, either within national or EU legal systems, was needed for such a procedure, as enrolment was on a voluntary basis and the participants consented to anonymous information collection as per the General Data Protection Regulation (Regulation (EU) 2018/679). Interviewees agreed to participate in the study voluntarily by self‐enrolling. They were informed that their answers would be published in a study. By completing and returning the questionnaire, they agreed to the inclusion of their data.

## Conflicts of Interest

The authors declare no conflicts of interest.

## Supporting information




**Supporting File 1**: vms371170‐sup‐0001‐SuppMat1.pdf

**Supporting File 2**: vms371170‐sup‐0002‐SuppMat2.docx

## Data Availability

The data that support the findings of this study are available from the corresponding author upon reasonable request.
