# Supplementary material for: Feeding and Husbandry Practices in Pet Ferrets (Mustela putorius furo): A Cross‐Sectional International Study
Source: Vet Med Sci. 2026 Aug 13;12(5):e71170. doi: 10.1002/vms3.71170 (PMC13472074; doi:10.1002/vms3.71170)
Supplement: Supplementary file 1 [file VMS3-12-e71170-s001.pdf]

QUESTIONNAIRE FOR FERRETS OWNERS

Hello, my name is Elena Dal Corso. I am a 5th year Veterinary Medicine student at Padua University working on a thesis on the management and nutrition of ferrets. I have developed the questionnaire below with easy questions for ferret owners. By filling it out, you will be making an important contribution to research on ferret management and help us learn more in this field that has still not received much attention.
The questionnaire is divided into four sections (owner data, animal data, management, nutrition). It should only take 20 minutes to fill out. In accordance with Personal Data Protection law D.Lgs 193/2003 and GDPR 2016/679, the data collected is anonymous, does not involve the diffusion of any personal information, and will be used for research purpose only. Thank you for your kind cooperation.

*Campo obbligatorio

OWNER DATA

1. 1. How old are you? *

Contrassegna solo un ovale.

- Under 18
- From 18 to 25
- From 26 to 35
- From 36 to 45
- From 46 to 55
- From 56 to 65
- Over 65

2. 2. Gender: *

Contrassegna solo un ovale.

- Male
- Female
- Other

3. 3. In which country do you live? *

4. 4. Whom is your household composed of? *

Contrassegna solo un ovale.

- I live alone
- I live with my partner
- I live in a household composed of more than two people
- I live with housemates
- Altro: ____________

5. 5. Select the members that compose your household from the ones listed: *

Seleziona tutte le voci applicabili.

- Children less than 4 years old
- Children from 4 to 14 years old
- Children more than 14 years old
- Pregnant women
- People older than 65 years
- Immunocompromised people (for example, people with chronic or neoplastic disease or those using immunosuppressing drugs)
- None of the above

6. 6. Is this your first experience as a ferret owner? *

Contrassegna solo un ovale.

- Yes, this is (these are) my first ferret (ferrets)
- No, I have already had other ferrets

7. 7. How many years of experience as a ferret owner do you have? *

Contrassegna solo un ovale.

- Less than 1
- From 1 to 5
- From 6 to 10
- From 11 to 20
- More than 20

8. 8. Select your category: *

Contrassegna solo un ovale.

- Pet ferret owner
- Ferret breeder
- Veterinarian and pet ferret owner
- Veterinarian and ferret breeder
- Veterinary Medicine student and pet ferret owner
- Veterinary Medicine student and ferret breeder

ANIMAL DATA

9. 1. How many ferrets do you own? *

Contrassegna solo un ovale.

- One *Passa alla domanda 11.*
- Two
- Three
- More than three

10. 1.a. If you own more than one ferret, has a new ferret been introduced in the group recently (in the last 2 months)? *

Contrassegna solo un ovale.

- Yes
- No

11. 2. Does/do your ferret(s) live(s) with: *

Seleziona tutte le voci applicabili.

- [ ] No other animals
- [ ] One or more dogs
- [ ] One or more cats
- [ ] One or more birds
- [ ] One or more reptiles
- [ ] One or more small mammals (for example hamsters, rabbits)

Altro: [ ] ____________________

12. 3. How old is your ferret? *

If you own more than one ferret, I now ask you to choose one of your ferrets and to answer this and the next questions referring only to this one.

Contrassegna solo un ovale.

- Less than 6 months
- From 6 months to 1 year
- From 2 to 3 years
- From 4 to 5 years
- From 6 to 8 years
- More than 8 years
- I don't know

13. 4. Select your ferret's coat color: *

Contrassegna solo un ovale.

- Champagne
- Cinnamon
- Chocolate
- Sable
- Black sable
- Black
- Albino
- Silver
- I don't know
- Altro: ____________________

14. 5. Select your ferret's coat color pattern: *

Contrassegna solo un ovale.

- Roan
- Siamese
- Standard
- Solid
- Self
- I don't know
- Altro: ____________________

15. 6. Select your ferret's coat length: *

Contrassegna solo un ovale.

- Short
- Mid angora
- Angora
- I don't know
- Altro: ____________________

16. 7. How did you get your ferret? *

Contrassegna solo un ovale.

- I adopted it from an association or from the last owner
- I bought it in a pet shop
- I bought it in a ferret breeding farm
- I bought it from a private owner
- I don’t know, it was a gift
- Altro: ____________________

17. 8. What is your ferret’s gender? *

Contrassegna solo un ovale.

- Intact male *Passa alla domanda 20.*
- Intact female *Passa alla domanda 20.*
- Neutered male
- Neutered female

18. 8.a. Select the sterilization method: *

Contrassegna solo un ovale.

- Surgical sterilization
- Chemical sterilization

19. 8.b. At which age was your ferret neutered? (Please, provide only the information related to the ferret you chose as reference) *

____

20. 9. How much does your ferret weigh? Remember to write the unit of measure if other than kg. (Please, provide only the information related to the ferret you chose as reference) *

____

21. 10. What is your ferret's length from the tip of nose to tip of tail? Remember to write the unit of measure if other than cm. (Please, provide only the information related to the ferret you chose as reference)

____

22. 11. How do you assess your ferret's body condition? *

Contrassegna solo un ovale.

- It is fit and normal weight
- It is underweight
- It is overweight
- It is obese

23. 12. How many hours per day does your ferret sleep on average? *

Contrassegna solo un ovale.

- Less than 8
- From 8 to 12
- From 12 to 16
- From 16 to 20
- More than 20

24. 13. When your ferret is not sleeping, how do you assess its level of activity? *

Contrassegna solo un ovale.

- It is very active and excited
- It is active, explores and plays
- It is quite calm and not very active
- It is inactive and doesn't move much

25. 14. How do you assess your ferret's usual faeces texture? *

Contrassegna solo un ovale.

- Liquid
- Soft
- Compact and well-shaped
- Hard

26. 15. Assess your ferret's coat quality by giving a score from 0 to 5 to every coat characteristic that is listed. 0 = absent, 5 = very present. *

Contrassegna solo un ovale per riga.

	0	1	2	3	4	5
Shine						
Density						
Presence of alopecia						
Presence of dandruff						
Greasiness						

27. 16. If there is alopecia, where is it distributed?

Contrassegna solo un ovale.

- On the tail
- On the limbs
- On the trunk
- On the head
- Altro: ______________________

MANAGEMENT

28. 1. How old was your ferret when you first received it? *

Contrassegna solo un ovale.

- Less than 6 months
- From 6 months to 1 year
- From 2 to 3 years
- From 4 to 5 years
- From 6 to 8 years
- More than 8 years
- I don’t know

29. 2. Where does your ferret live? *

Contrassegna solo un ovale.

- Indoors
- Outdoors
- Both indoors and outdoors

30. 3. Where does your ferret usually stay? *

Contrassegna solo un ovale.

- In a cage from which it never goes out
- In a cage from which it can go out and access a dedicated area
- In a cage from which it can go out and move freely in the house
- In a room completely dedicated to it
- It is always free in the house
- Altro: ______________________

31. 4. How much time does your ferret spend outside its cage on average? *

Contrassegna solo un ovale.

- It is always outside because it does not live in a cage
- More than 8 hours per day
- From 6 to 8 hours per day
- From 4 to 6 hours per day
- From 2 to 4 hours per day
- From 1 to 2 hours per day
- Less than 1 hour per day
- It never goes outside its cage

32. 5. What are the cage's features? *

Contrassegna solo un ovale.

- It has multiple floors
- It has only one floor
- I don't have a cage

33. 6. Which objects are there inside the cage or in the area where your ferret lives? Select everything that is present (it is possible to select multiple choices). *

Seleziona tutte le voci applicabili.

- [ ] Litter
- [ ] Food bowl
- [ ] Water bowl
- [ ] Drinking bottle
- [ ] Hammock
- [ ] Hiding place
- [ ] Rope
- [ ] Ladder or ramp
- [ ] Toys
- [ ] Tunnel or maze
- [ ] Dig box

Altro: [ ] ____________________

34. 7. Where does your ferret prefer to drink from? *

Contrassegna solo un ovale.

- Drinking bottle
- Bowl
- Both
- Altro: ______________________

35. 8. How often do you change the water? *

Contrassegna solo un ovale.

- More than once a day
- Once a day
- Less than once a day
- I don’t know, when I remember

36. 9. What kind of litter do you use? *

Contrassegna solo un ovale.

- Vegetal material (for example corncob)
- Clumping litter (for example cat litter)
- Paper
- Altro: ______________________

37. 10. How often do you change the litter? *

Contrassegna solo un ovale.

- More than once a day
- Once a day
- Every other day
- Twice a week
- Once a week
- Less than once a week

38. 11. How often do you clean your ferret's ears? *

Contrassegna solo un ovale.

- More than once a week
- Once a week
- Once every two weeks
- Once a month
- Less than once a month
- Never

39. 12. How often do you clean your ferret's coat? *

Contrassegna solo un ovale.

- More than once a week
- Once a week
- Once every two weeks
- Once a month
- Less than once a month
- Never

40. 13. How often do you cut your ferret's claws? *

Contrassegna solo un ovale.

- More than once a week
- Once a week
- Once every two weeks
- Once a month
- Less than once a month
- Never

41. 14. How many hours do you spent interacting with your ferret in a day? *

Contrassegna solo un ovale.

- Less than 0.5
- From 0.5 to 1
- From 1 to 2
- From 2 to 4
- More than 4

42. 15. Has your ferret ever displayed any of these behaviours? Select one or more behaviours you have noticed recently or in the past in your ferret from the ones listed below. *

Seleziona tutte le voci applicabili.

- Defecate or urinate outside the litter
- Bite or attack the owner
- Bite or attack other people
- Bite or attack other ferrets
- Bite or hurt itself
- Bite or attack objects or the cage
- Hide some food
- Pacing (walking back and forth many times) the same path
- Scratching in a compulsive way
- I have never noticed any of these behaviours in my ferret

43. 16. Did you train your ferret? Choose your option: I trained my ferret to... *

Seleziona tutte le voci applicabili.

- [ ] Use the litter
- [ ] Come when I call its name
- [ ] Learn some exercises
- [ ] I haven't trained my ferret

Altro: [ ] ____________________

44. 17. Do you vaccinate your ferret? *

Contrassegna solo un ovale.

- ( ) Yes, regularly
- ( ) Yes, but not regularly
- ( ) No, never

45. 18. Do you treat your ferret with antiparasitic products? *

Contrassegna solo un ovale.

- ( ) Yes, regularly
- ( ) Yes, but not regularly
- ( ) No, never

46. 19. Do you treat your ferret with preventive medicine for heartworm disease? *

Contrassegna solo un ovale.

- ( ) Yes, regularly
- ( ) Yes, but not regularly
- ( ) No, never

47. 20. How often do you take your ferret to the vet for a check-up? *

Contrassegna solo un ovale.

- More than twice a year
- Twice a year
- Once a year
- Less than once a year
- I take it to the vet only when I see that it is not ok

48. 21. Has your referring vet ever diagnosed any of the diseases below in your ferret? Select the disease(s) diagnosed. (Please, provide only the information related to the ferret you chose as reference) *

Seleziona tutte le voci applicabili.

- Cardiac disease
- Kidney disease
- Urinary tract disease (for example, stones)
- Reproductive system disease
- Adrenal glands tumor
- Insulinoma
- Other tumors
- Blindness
- Deafness
- Odontolithiasis (dental tartar)
- Gastrointestinal parasites
- Ectoparasites
- Heartworm disease
- No disease has ever been diagnosed in my ferret

Altro: ____________________

49. 22. Has your ferret ever had a dental cleaning (tartar removal)? *

Contrassegna solo un ovale.

- Yes, more than once
- Yes, once
- No, but maybe it should
- No, it doesn’t have tartar on its teeth

50. 23. Do you clean your ferret’s teeth? *

Contrassegna solo un ovale.

- Yes, once or more times a day
- Yes, about once every 2-3 days
- Yes, about once a week
- Yes, but less often than once a week
- No, never

51. 24. Who or what is your main source of information on your ferret's healthcare management? *

Contrassegna solo un ovale.

- Books
- Websites and blogs
- Groups on social media
- Other ferrets owners that you know
- Ferret breeders
- Associations
- Pet shop salesperson
- Veterinarian
- Exotic animals specialist veterinarian
- I don't refer to any particular source of information

52. 25. Who or what is your main source of information on the general management of your ferret? *

Contrassegna solo un ovale.

- Books
- Websites and blogs
- Groups on social media
- Other ferrets owners that you know
- Ferret breeders
- Associations
- Pet shop salesperson
- Veterinarian
- Exotic animals specialist veterinarian
- I don't refer to any particular source of information

NUTRITION

53. 1. Do you give your ferret dry feed? *

Answer “yes” if you usually give dry feed to your ferret during the week, otherwise answer “no” if you use dry feed less often or if you never use it.

Contrassegna solo un ovale.

- Yes
- No *Passa alla domanda 60.*

54. 1.a. What kind of dry feed do you use? *

Contrassegna solo un ovale.

- Ferret dry feed
- Cat dry feed
- Dog dry feed
- Dry feed for species other than cats or dogs
- Both ferret dry feed and dry feed for cats, dogs or other species

55. 1.b. What brand of dry feed do you use? Write brand and name of the feed. *

If you use more than one dry feed, write the brand and name of every dry feed that you use separating them with ";".

56. 1.c. How often do you change brand or kind of feed? *

Contrassegna solo un ovale.

- Never, I always use the same feed(s)
- Once or twice a year
- From three to six times per year
- Every month
- Every two weeks
- Every week

57. 1.d. How do you give the dry feed to your ferret? *

Contrassegna solo un ovale.

- The feed is always available *Passa alla domanda 59.*
- I give controlled amounts of feed
- I give variable amounts of feed *Passa alla domanda 59.*

58. 1.e. How do you control the amount of dry feed you give your ferret? *

Contrassegna solo un ovale.

- By eye
- I use a measuring cup
- I weigh it on a kitchen scale
- Altro: ____________________

59. 1.f. When do you give dry feed to your ferret? *

Contrassegna solo un ovale.

- The feed is always available
- I give the feed at fixed times in the day
- I give the feed at variable times in the day

60. 2. Do you give your ferret industrial wet feed (e.g. gravy preparations, chunk-in-loaf preparations etc.)? Wet feed does not include fresh meat, eggs or other homemade food (see question n. 3) *

Answer “yes” if you usually give wet feed to your ferret during the week, otherwise answer “no” if you use wet feed less often or if you never use it.

Contrassegna solo un ovale.

- Yes
- No *Passa alla domanda 67.*

61. 2.a. What kind of wet feed do you use? *

Contrassegna solo un ovale.

- Ferret wet feed
- Cat wet feed
- Dog wet feed
- Wet feed for species other than cats and dogs
- Both ferret wet feed and wet feed for cats, dogs or other species

62. 2.b. What brand of wet feed do you use? Write brand and name of the feed. *

If you use more than one wet feed, write the brand and name of every wet feed that you use separating them with “;”.

63. 2.c. How often do you change brand or kind of feed? *

Contrassegna solo un ovale.

- Never, I always use the same feed(s)
- Once or twice a year
- From three to six times per year
- Every month
- Every two weeks
- Every week

64. 2.d. How do you give the wet feed to your ferret? *

Contrassegna solo un ovale.

- The feed is always available *Passa alla domanda 66.*
- I give controlled amounts of feed
- I give variable amounts of feed *Passa alla domanda 66.*

65. 2.e. How do you control the amount of wet feed you give to your ferret? *

Contrassegna solo un ovale.

- By eye
- I use a measuring cup
- I weigh it on a kitchen scale

66. 2.f. When do you give wet feed to your ferret? *

Contrassegna solo un ovale.

- The feed is always available
- I give the feed at fixed times in the day
- I give the feed at variable times in the day

67. 3. Do you give your ferret homemade food? *

Answer “yes” if you usually give homemade food to your ferret during the week, otherwise answer “no” if you use homemade food less often or if you never use it.

Contrassegna solo un ovale.

- Yes
- No *Passa alla domanda 76.*

68. 3.a. Which homemade foods do you usually give your ferret during the week? *

Seleziona tutte le voci applicabili.

- Raw meat and/or organs
- Cooked meat
- Fish
- Bones
- Eggs
- Dairy products
- Cereals or carbohydrates sources (pasta, rice, bread...)
- Nuts
- Vegetables
- Fruit

Altro: ______________________

69. 3.b. Which kind of raw or cooked meat do you usually give your ferret during the week? *

Seleziona tutte le voci applicabili.

- [ ] Chicken
- [ ] Turkey
- [ ] Quail
- [ ] Duck
- [ ] Goose
- [ ] Guineafowl
- [ ] Pork
- [ ] Beef
- [ ] Horse
- [ ] Rabbit
- [ ] No one

Altro: [ ] ______________________

70. 3.c. Which organs do you usually give your ferret during the week? *

Seleziona tutte le voci applicabili.

- [ ] Liver
- [ ] Kidney
- [ ] Heart
- [ ] Lungs
- [ ] Stomach or intestines
- [ ] Brain
- [ ] No one

Altro: [ ] ______________________

71. 3.d. Which kind of fish do you usually give your ferret during the week? *

Seleziona tutte le voci applicabili.

- [ ] Salmon
- [ ] Trout
- [ ] Pilchard
- [ ] Cod
- [ ] Flounder or sole
- [ ] Tuna
- [ ] Molluscs
- [ ] None

Altro: [ ] ______________________

72. 3.e. For every kind of food listed, select a percentage in relation to the total amount of homemade food you usually give your ferret during a week (for example, in a week I give my ferret 70% raw meat and/or organs, 20% bones and 10% vegetables). The sum of the selected percentages must be 100%. If you usually do not use that kind of food, select 0%. *

Contrassegna solo un ovale per riga.

	0%	10%	20%	30%	40%	50%	60%	70%
Raw meat and/or organs	◯	◯	◯	◯	◯	◯	◯	◯
Cooked meat	◯	◯	◯	◯	◯	◯	◯	◯
Fish	◯	◯	◯	◯	◯	◯	◯	◯
Bones	◯	◯	◯	◯	◯	◯	◯	◯
Eggs	◯	◯	◯	◯	◯	◯	◯	◯
Dairy products	◯	◯	◯	◯	◯	◯	◯	◯
Cereals or carbohydrates sources (pasta, rice, bread...)	◯	◯	◯	◯	◯	◯	◯	◯
Nuts	◯	◯	◯	◯	◯	◯	◯	◯
Vegetables	◯	◯	◯	◯	◯	◯	◯	◯
Fruit	◯	◯	◯	◯	◯	◯	◯	◯
Other	◯	◯	◯	◯	◯	◯	◯	◯

73. 3.f. How do you give your ferret homemade food? *

Contrassegna solo un ovale.

◯ The food is always available *Passa alla domanda 75.*

◯ I give controlled amounts of food

◯ I give variable amounts of food *Passa alla domanda 75.*

74. 3.g. How do you control the amount of homemade food you give your ferret? *

Contrassegna solo un ovale.

- By eye
- I use a measuring cup
- I weigh it on a kitchen scale

75. 3.h. When do you give your ferret homemade food? *

Contrassegna solo un ovale.

- The food is always available
- I give the food at fixed times in the day
- I give the food at variable times in the day

76. 4. Do you give your ferret whole preys? *

Answer "yes" if you usually give whole preys to your ferret during the week, otherwise answer "no" if you use whole preys less often or if you never use it.

Contrassegna solo un ovale.

- Yes
- No *Passa alla domanda 79.*

77. 4.a. Which kind of preys do you usually give your ferret during the week? *

Seleziona tutte le voci applicabili.

- [ ] Chicks
- [ ] Rats or mice
- [ ] Frogs
- [ ] Quails
- [ ] Bunnies
- [ ] Fish
- [ ] Insects

Altro: [ ] ____________________

78. 4.b. How often in a week do you give whole preys to your ferret? *

Contrassegna solo un ovale.

- 7 days a week
- 5 or 6 days a week
- 3 or 4 days a week
- 1 or 2 days a week
- Less than once a week

79. 5. Do you give your ferret snacks? *

Contrassegna solo un ovale.

- No, I never give it snacks *Passa alla domanda 82.*
- Yes, I use its usual feed as a snack
- Yes, I use homemade food as a snack
- Yes, I give some industrial snacks
- Yes, I use both homemade food and industrial snacks

80. 5.a. What kind of snacks do you use? Select the kind of snacks you usually give your ferret during the week. *

Seleziona tutte le voci applicabili.

- [ ] Fruit
- [ ] Vegetables
- [ ] Nuts
- [ ] Raisins
- [ ] Meat and/or organs
- [ ] Fish
- [ ] Eggs
- [ ] Dairy products
- [ ] Cereals or carbohydrate sources
- [ ] Industrial snacks for ferrets
- [ ] Industrial snacks for cats, dogs or other species
- [ ] Biscuits or cookies for humans
- [ ] Leftovers
- [ ] Malt paste (GimPet, GimCat)

Altro: [ ] ____________

81. 5.b. How often do you give your ferret snacks? *

Contrassegna solo un ovale.

- ( ) More than once a day
- ( ) Once a day
- ( ) Every other day
- ( ) Twice a week
- ( ) Once a week
- ( ) Less than once a week

82. 6. For every kind of food listed, select a percentage in relation to the total amount of food you usually give your ferret during a week (for example, in a week I give my ferret 40% dry food, 40% wet food and 20% homemade food). The sum of the selected percentages must be 100%. If you usually do not use that kind of food, select 0%. *

Contrassegna solo un ovale per riga.

	0%	10%	20%	30%	40%	50%	60%	70%	8(
Dry feed									
Wet feed									
Homemade food									
Whole preys									
Snacks									

83. 7. In your opinion, what kind of food does your ferret like most (consider every kind of food you usually give your ferret, including snacks)? *

84. 8. How often do you give your ferret its favorite food during a week? *

Contrassegna solo un ovale.

- 7 days a week
- 5 or 6 days a week
- 3 or 4 days a week
- 1 or 2 days a week
- Less than once a week

85. 9. What kind of supplements do you give your ferret? *

Seleziona tutte le voci applicabili.

- [ ] I do not use any kind of supplement
- [ ] Supplements for coat
- [ ] Supplements for joints
- [ ] Antioxidant supplements
- [ ] Supplements that promote gastrointestinal motility and fur expulsion
- [ ] Vitamin and mineral supplements
- [ ] Supplements for balancing homemade diet

Altro: [ ] ______________________

86. 10. Have you ever noticed an adverse reaction to food in your ferret (any symptom like fatigue, diarrhea, or vomiting) after giving it any particular food? If so, write the name of the food you think was responsible (chicken meat, for example). *

____

87. 11. Who or what is your main source of information about the nutrition of your ferret? *

Contrassegna solo un ovale.

- ( ) Books
- ( ) Websites and blogs
- ( ) Groups on social media
- ( ) Other ferrets owners that you know
- ( ) Ferret breeders
- ( ) Associations
- ( ) Pet shop salesperson
- ( ) Veterinarian
- ( ) Exotic animals specialist veterinarian
- ( ) I don't refer to any particular source of information

88. That was the last question of the questionnaire! Are you reasonably sure to have sent in your completed questionnaire correctly? If you want to change one or more answers that you gave, you can do so now clicking on the “Back” button. You won’t be able to modify your answers after sending in your form. We thank you for dedicating so much time. Your cooperation is precious. *

Contrassegna solo un ovale.

- Yes
- No

Questi contenuti non sono creati né avallati da Google.

Google Moduli
