# Supplementary material for: Feeding and Husbandry Practices in Pet Ferrets (Mustela putorius furo): A Cross‐Sectional International Study
Source: Vet Med Sci. 2026 Aug 13;12(5):e71170. doi: 10.1002/vms3.71170 (PMC13472074; doi:10.1002/vms3.71170)
Supplement: Supplementary file 2 [file VMS3-12-e71170-s002.docx]

Supplementary file 2 Fresh foods, snacks, and supplements provided to ferrets enrolled in the study (n = 495), stratified by country. Only items showing significant differences among countries (p < 0.05) are reported. Results are expressed as percentages of participants; chi-squared (χ²) and p-values are provided.

|  | Belgium | France | Italy | UK | US |  |
| --- | --- | --- | --- | --- | --- | --- |
| Foods | | | | | | |
| RMBD and organs | 30.8 | 35.5 | 44.4 | 23.1 | 30.9 | $x^{2}$= 27.3;  p = 0.007 |
| Fish | 7.7 | 16.7 | 1.9 | 0.0 | 10.3 | $x^{2}$= 51.6;  p = 0.002 |
| Bones | 15.4 | 11.8 | 14.8 | 10.3 | 11.8 | $x^{2}$ = 62.2;  p < 0.001 |
| Eggs | 38.5 | 38.2 | 33.3 | 23.1 | 32.4 | $x^{2}$ = 24.3;  p = 0.02 |
| Dairy products | 0.0 | 0.5 | 1.9 | 0.0 | 0.0 | $x^{2}$ = 101.1;  p = 0.03 |
| Meat types | | | | | | |
| Chicken | 38.5 | 35.5 | 42.6 | 25.6 | 30.9 | $x^{2}$ = 25.5;  p = 0.02 |
| Turkey | 7.7 | 24.7 | 29.6 | 20.5 | 14.7 | $x^{2}$ = 25.0;  p = 0.03 |
| Quail | 15.4 | 14.0 | 32.1 | 2.6 | 16.2 | $x^{2}$ = 47.1;  p < 0.001 |
| Duck | 30.8 | 12.4 | 11.7 | 17.9 | 19.1 | $x^{2}$ = 36.4;  p = 0.005 |
| Goose | 0.0 | 1.1 | 2.5 | 2.6 | 5.9 | $x^{2}$ = 108.6;  p = 0.001 |
| Pork | 15.4 | 4.3 | 3.1 | 15.4 | 8.8 | $x^{2}$ = 63.7;  p < 0.001 |
| Beef | 7.7 | 12.9 | 4.3 | 25.6 | 11.8 | $x^{2}$ = 65.4;  p < 0.001 |
| Rabbit | 30.8 | 17,7 | 30,9 | 15.4 | 17.6 | $x^{2}$ = 31.3;  p = 0.003 |
| Offals | | | | | | |
| Liver | 23.1 | 23.7 | 26.5 | 12.8 | 22.1 | $x^{2}$ = 37.1;  p = 0.002 |
| Kidney | 15.4 | 9.1 | 11.1 | 12.8 | 19.1 | $x^{2}$ = 66.9;  p < 0.001 |
| Heart | 30.8 | 18.3 | 29.0 | 20.5 | 23.5 | $x^{2}$ = 40.1;  p = 0.001 |
| Lungs | 0.0 | 4.3 | 14.8 | 10.3 | 4.4 | $x^{2}$ = 65.0;  p = 0.001 |
| Brain | 0.0 | 3.8 | 2.5 | 2.6 | 8.8 | $x^{2}$ = 79.2;  p = 0.001 |
| Fish | | | | | | |
| Salmon | 23.1 | 19.4 | 2.5 | 2.6 | 16.2 | $x^{2}$ = 49.3;  p < 0.001 |
| Preys | | | | | | |
| Chicks | 30.8 | 28.0 | 6.2 | 30.8 | 5.9 | $x^{2}$ = 71.5;  p < 0.001 |
| Mice or rats | 15.4 | 13.4 | 3.7 | 33.3 | 5.9 | $x^{2}$ = 76.5;  p < 0.001 |
| Quails | 0.0 | 12.4 | 19.1 | 12.8 | 7.4 | $x^{2}$ = 44.7;  p = 0.001 |
| Snacks | | | | | | |
| Industrial snacks for ferrets | 53.8 | 22.0 | 10.5 | 10.3 | 27.9 | $x^{2}$ = 29.4;  p = 0.02 |
| Malt paste | 0.0 | 9.7 | 45.1 | 2.6 | 2.9 | $x^{2}$ = 112.4;  p < 0.001 |
| Supplements | | | | | | |
| Coat | 23.1 | 30.1 | 26.5 | 7.7 | 19.1 | $x^{2}$ = 29.0;  p = 0.009 |
| Gastro intestinal | 15.4 | 8.1 | 22.8 | 5.1 | 2.9 | $x^{2}$ = 32.2;  p = 0.03 |
| General vitamin and mineral | 23.1 | 13.4 | 35.8 | 10.3 | 11.8 | $x^{2}$ = 46.7;  p < 0.001 |
